# Immune checkpoint blockade in glioblastoma: from tumor heterogeneity to personalized treatment

**DOI:** 10.1172/JCI163447

**Published:** 2023-01-17

**Authors:** Víctor A. Arrieta, Crismita Dmello, Daniel J. McGrail, Daniel J. Brat, Catalina Lee-Chang, Amy B. Heimberger, Dhan Chand, Roger Stupp, Adam M. Sonabend

**Affiliations:** 1 Department of Neurological Surgery, Northwestern University Feinberg School of Medicine, Chicago, Illinois, USA.; 2 Program of Combined Studies in Medicine (PECEM), Faculty of Medicine, National Autonomous University of Mexico, Mexico City, Mexico.; 3 Northwestern Medicine Malnati Brain Tumor Institute of the Lurie Comprehensive Cancer Center, Northwestern University Feinberg School of Medicine, Chicago, Illinois, USA.; 4 Center for Immunotherapy and Precision Immuno-Oncology and; 5 Lerner Research Institute, Cleveland Clinic, Cleveland, Ohio, USA.; 6 Department of Pathology, Northwestern University Feinberg School of Medicine, Chicago, Illinois, USA.; 7 Agenus Bio, Lexington, Massachusetts, USA.; 8 Department of Neurology, Northwestern University Feinberg School of Medicine, Chicago, Illinois, USA.

## Abstract

Immune checkpoint blockade (ICB) has revolutionized modern cancer therapy, arousing great interest in the neuro-oncology community. While several reports show that subsets of patients with glioma exhibit durable responses to immunotherapy, the efficacy of this treatment has not been observed for unselected patient populations, preventing its broad clinical implementation for gliomas and glioblastoma (GBM). To exploit the maximum therapeutic potential of ICB for patients with glioma, understanding the different aspects of glioma-related tumor immune responses is of critical importance. In this Review, we discuss contributing factors that distinguish subsets of patients with glioma who may benefit from ICB. Specifically, we discuss (a) the complex interaction between the tumor immune microenvironment and glioma cells as a potential influence on immunotherapy responses; (b) promising biomarkers for responses to immune checkpoint inhibitors; and (c) the potential contributions of peripheral immune cells to therapeutic responses.

## Introduction

Despite the remarkable success of immune checkpoint blockade (ICB) seen in many cancer types, the same efficacy is not observed in glioblastoma (GBM), underscoring the importance of understanding the variety of factors that dictate immune responses for this type of brain tumor. Unfortunately, all immunotherapies tested to date have failed to improve clinical outcomes in unselected cohorts of patients with GBM. For example, three controlled phase III trials assessing nivolumab, an anti–programmed cell death protein 1 (anti–PD-1) therapy, have failed to demonstrate a survival advantage in patients with GBM. In the CheckMate 143 clinical trial, median overall survival was comparable between nivolumab-treated and bevacizumab-treated control patients (1). Similarly, in the CheckMate 498 clinical study, PD-1 blockade in combination with radiotherapy in newly diagnosed GBM patients with unmethylated *MGMT* promoter failed to meet its primary endpoint of improvement in survival, as the cohort that received the alkylating chemotherapy temozolomide (TMZ) plus radiotherapy showed a longer overall survival (2). Lastly, nivolumab in combination with TMZ and radiotherapy was found not to be superior to TMZ, radiotherapy, and placebo in newly diagnosed GBM patients with methylated *MGMT* promoter (3). Despite these negative results, several studies and case reports have shown that some patient subsets demonstrate durable radiographic responses and prolonged survival following this form of ICB (1, 4–9). Clinical studies have evaluated markers of immune response after anti–PD-1 therapy administered in the neoadjuvant setting (5, 10, 11). In these trials, neoadjuvant PD-1 blockade induced clinical benefit and immunological responses in patients with recurrent GBM as compared with adjuvant immunotherapy (5, 12). In light of these observations, a key question when considering immunotherapy for the treatment of GBM is how to identify patients a priori who are likely to respond, and what variables can influence efficacy. Though responsive GBM patients might be a minority in clinical studies evaluating ICB as monotherapy, the accurate identification of these individuals could provide them with a meaningful clinical benefit.

In this Review, we provide perspectives on the use of ICB in gliomas and GBM, specifically emphasizing the biological aspects of such brain tumors and the factors that influence clinical response. These include the diversity of phenotypes in GBM that are associated with differences in the immune cell composition and functionality within each tumor; promising predictive biomarkers for ICB; and the generation of an adequate peripheral immune response during treatment.

## Tumor-immune crosstalk may influence the ICB response

Although the brain was once considered “immune privileged,” recent studies indicate that “immune peculiar” may be a more accurate descriptor. Under non-disease conditions, the brain is largely immunologically quiescent, with the blood-brain barrier (BBB) limiting the infiltration of circulating immune cells (13). Instead, embryonically derived resident microglial cells serve as resident macrophages to survey the neural environment (14), with further immunosurveillance provided by a specialized lymphatic drainage system (15). Cancer and other inflammatory conditions can permeate the BBB, allowing for infiltration of T cells and other peripheral leukocytes (13, 16, 17). Together, these features translate to a unique tumor microenvironment consisting of 20%–40% immune cells, the majority of which are myeloid cells with varying ratios of tissue-resident microglia and bone marrow–derived macrophages from circulation (18). Infiltration of both monocyte-derived macrophages and lymphocytes has been shown to be higher in *IDH*-wild-type tumors, whereas the immune compartment of *IDH*-mutant tumors was nearly exclusively microglia. TMZ can reduce the numbers of antiinflammatory myeloid-derived suppressor cells (MDSCs), although the prevalence of MDSCs increases in late-stage tumors, where current ICB efforts have been focused (19, 20). MDSC origin and function can also vary in GBM by the sex of the patient (21). Other components of the tumor microenvironment, most notably astrocytes, may also play a role in the modulation of immune responses in GBM (22).

Classically, oncogenic signaling in cancer cells has been attributed to tumor cell genetic alterations such as oncogene activation and/or loss of tumor suppressors (23). Recently, several groups have reported robust associations between tumor cell phenotypes and inflammatory features of the tumor microenvironment in gliomas (6, 24–26). While drawing causal relationships based on associations is challenging, studies have suggested that immune cells and non-neoplastic cells within the tumor microenvironment influence tumor cell phenotype (24, 27, 28). On the other hand, tumor cells might also modulate the recruitment and phenotype of myeloid cells, microglia, and reactive glial cells (25, 29). This dynamic crosstalk between tumor and immune cells and other non-neoplastic cells ultimately shapes and contributes to the variable inflammatory phenotypes and heterogeneity found in the tumor microenvironment encountered across gliomas.

Glioma cell phenotype and wider tumor-immune microenvironment differences should be considered for personalizing immunotherapy for gliomas. In this regard, valuable efforts have led to the discovery of multiple cellular states with critical implications in the composition and function of the tumor immune microenvironment (24, 25, 27). Through several analytical approaches, it has been demonstrated that a subpopulation of GBM cells leverages neurodevelopmental stem cell programs as a central axis to sustain tumor growth (30–32). Additionally, an inflammatory GBM phenotype associated with abundant immune cells has been described across clinical cohorts (25, 27, 30). By integration of single-cell RNA-Seq and bulk RNA-Seq data from human GBMs, four cellular states were proposed: an astrocyte-like (AC-like) program, an oligodendrocyte precursor cell–like (OPC-like) program, a neural progenitor cell–like (NPC-like) program, and a mesenchymal-like (MES-like) program (31). The AC-like, OPC-like, and NPC-like programs were associated with neurodevelopmental genes linked to progenitor cells of neuronal/glial lineages (31). Notably, the MES-like program did not resemble progenitor or stem cell–like phenotypes but rather was associated with high proportions of macrophages, microglia, and cytotoxic T cells (24).

Similarities were also found in a different classification that mapped GBM stem cells in a transcriptional gradient along a “Developmental” program and an “Injury Response” program (30). Tumor cells in the Developmental state resembled progenitor cells associated with neurodevelopmental programs characterized by substantial levels of proliferation. At the other end of the spectrum, GBM cells displaying the Injury Response state had gene expression signatures reminiscent of an inflammatory wound response that included genes related to NF-κB, signal transducer and activator of transcription (STAT) signaling, and reactive astrocytes. Notably, inflammatory cytokines such as TNF-α, IL-1α, and complement component 1q (C1q), known to be secreted by microglia (33), were able to shift the gene expression program of GBM from a Developmental to an inflammatory Injury Response program, suggesting a malleable capacity to transcriptionally adapt to various microenvironmental cues. Compared with previous molecular classifications, tumor cells exhibiting an inflammatory wound response program showed a high overlap with The Cancer Genome Atlas (TCGA) mesenchymal subtype and the MES-like cell state, underscoring the consistency in detecting an immune-associated tumor profile of GBM.

A third study used an integrative approach of spatial transcriptomics, metabolomics, and proteomics to classify the cellular heterogeneity of human GBM (27). By analysis of a cohort of spatial data sets from GBM tissues from different patients, five transcriptional programs were proposed, including three related to progenitors of neuronal/glial lineages, referred to as Neuronal Development, Spatial OPC, and Radial Glia. The remaining two programs were related to reactive states, including a glycolytic/hypoxic module named “Reactive Hypoxia” and an inflammatory module termed “Reactive Immune.” These transcriptional programs were consistent with the two subgroups (one hypoxia dependent and the other hypoxia independent) described for the MES-like cell state (31). Remarkably, this study suggested that the tumor microenvironment can dictate the cellular phenotype and genetic status of glioma cells, leading to genomic instability. This was illustrated by the acquisition of copy number alterations such as loss of chromosomes 15p and 14q and gain of chromosome 7 by tumor cells related to a surrounding hypoxic environment. On the other hand, the Reactive Immune program was characterized by enrichment of IFN-γ signaling, expression of genes related to antigen presentation (*HLA-DRA*, *HLA-A*, *HLA-B*), enrichment of AC- and MES-like cells, and transcription of genes linked to reactive astrocytes. Notably, tumor regions of the Reactive Immune program contained abundant HLA-DR^+^ myeloid cells and T cells, highlighting the close association of an inflamed tumor cell phenotype with immune cell infiltration. In parallel, using transgenic mouse glioma models, our group reported that the absence of CD8^+^ T cells during gliomagenesis led to increased tumor infiltration of Iba1^+^ and CD11b^+^ myeloid cells, increased tumoral expression of phosphorylated ERK, increased genomic instability, and a proinflammatory tumor phenotype (28). Notably, recent evidence indicates that the ERK1/2 cascade of the MAPK pathway positively regulates the Reactive Immune program, the TCGA mesenchymal subtype, and the Injury Response program (27, 34). Indeed, loss-of-function mutations and deletions in *NF1*, a negative regulator of the MAPK pathway, are genetic alterations strongly associated with the TCGA mesenchymal subtype and the MES-like cell state (25, 31).

The close interaction between immune cells and tumor cells driving the emergence of an immunogenic GBM cell phenotype has been illustrated as an important feature of this type of brain cancer (24). Interaction analysis between ligands and receptors showed that through secretion of cytokines such as oncostatin M, macrophages can induce the MES-like phenotype in GBM cells, which was characterized by high expression of MHC class I and II–associated genes and increased susceptibility to T cell killing. This evidence builds on the notion that GBM evolves following a neurodevelopmental hierarchy and undergoes dynamic adaptations to a more inflammatory phenotype in response to the signals of an immune-enriched tumor microenvironment, including the upregulation of antigen-presenting molecules. Furthermore, these studies highlight the fact that the transcriptional identity of a subset of GBMs is not determined exclusively by the DNA somatic alterations but is governed by the dynamic communication with the immune tumor microenvironment during tumor progression (24, 30).

**Figure 1:**
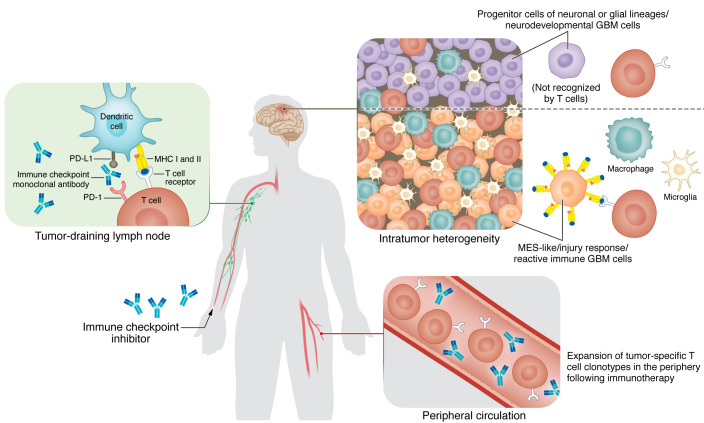
Dynamic crosstalk between tumor and immune cells as a potential contributor to an enhanced response to immune checkpoint blockade. Different transcriptional programs are defined by the abundance of immune cells such as macrophages, microglia, and T cells as well as the immunogenicity of tumor cells that includes the expression of MHC class I and II. In addition, the generation of a peripheral immune response following immune checkpoint inhibitors is a critical component of a successful therapeutic response.

The clinical importance of defining different phenotypes in GBM lies in the tumor’s intrinsic vulnerabilities and signaling dependencies associated with each transcriptional program that could be exploited therapeutically (30). Whether these types of GBMs are labeled as MES-like cells, Injury Response, or Reactive Immune, the clinical relevance of these classifications is that they highlight an inflammation-related phenotype of GBM that might be more suitable for immunotherapeutic approaches. The ability of peripheral and brain immune cells to induce an immunogenic tumor cell phenotype might also be an indicator of the strength of the patient’s immune system to mount an effective response during immunotherapy. Considering the immunological status of TCGA mesenchymal tumors and the poor prognosis compared with all other subtypes (25), this subset of tumors could be treated with therapies more suitable to their phenotype and immune cell content. Nonetheless, further investigation in preclinical models and patients with GBM will be able to confirm the susceptibility of this subset of inflamed tumors to immunotherapies and the associated biomarkers. A summary of evidence illustrating the crosstalk between tumor and immune cells is shown in Figure 1.

## Potential biomarkers to evaluate ICB response

Although predictive biomarkers for response to immune checkpoint inhibitors have been established for several cancers, they are limited to immunogenic tumor types, and most do not seem to be useful in gliomas. Biomarkers of response to ICB described for other cancers include programmed death ligand 1 (PD-L1) positivity on tumor and immune cells, expression of an IFN-γ gene signature, the status of the tumor immune microenvironment, T cell infiltration, microsatellite instability, high tumor mutational burden (TMB), and DNA mismatch repair (dMMR) defects, among others (35–37). The frequency of these biomarkers in GBM is very low and is restricted to small subsets of tumors, which partially explains the lack of response for most glioma patients (38–41). PD-L1 expression is heterogeneous and infrequent within tumors and in peripheral immune cells of glioma patients (39, 42). Particularly, expression of PD-L1 is lower in glioma-infiltrating monocyte-derived macrophages (MDMs) than in MDMs that infiltrate brain metastases (43). This may account for the lack of association between PD-L1 expression and survival in clinical trials evaluating ICB for GBM (2, 3, 41). Likewise, expression of the T cell–inflamed/IFN-γ gene signature has also shown no association with ICB outcomes in GBM (44).

Considering the multiple factors involved in an effective immune and clinical response following ICB, a multicomponent panel including clinical, genomic, and transcriptomic variables has been developed to improve the accuracy of predictive biomarkers of responses to immunotherapies in other cancer patients such as those with melanoma (45). Notably, in high-grade serous ovarian cancers, which tend to be characterized as poorly immunogenic with limited response to ICB, the use of a gene network demonstrated superior predictive power for response to immunotherapy as compared with single-gene or single-biomarker approaches (46). Therefore, rather than use of a single biomarker, the integration of multiple biomarkers specific to gliomas will likely be needed to accurately identify those patients who are likely to respond to immunotherapies. Below, we discuss the biomarkers under investigation for predicting response to ICB in gliomas.

### TMB and response to ICB.

Currently, the only approved indication for treatment of GBM with ICB is for recurrent disease that harbors a high TMB (>10 mutations per megabase). However, this approval arose from pan-cancer trials that did not include any patients with GBM (47). One meta-analysis failed to find evidence of improved response to ICB in gliomas with high TMB (48), and one clinical study suggests that recurrent GBM tumors with a very low TMB may be more responsive to immunotherapy (49). A recent analysis of over a thousand cancer patients treated with these immunotherapies revealed that clonal TMB was the most robust predictor for response to immune checkpoint inhibitors, whereas subclonal TMB was not significantly associated with response. Furthermore, 4 of 20 mutational signatures that predicted the formation of immunogenic epitopes, including mutational processes related to UV light, APOBEC, tobacco, and *POLE* (encoding DNA polymerase E), were associated with a higher likelihood of response to ICB (50). Whereas these mutational signatures are frequent in cancer patients who exhibit clinical responses to ICB, they are clearly not prevalent in the majority of patients with GBM (51). Nevertheless, a benefit from anti–PD-1 and anti–PD-L1 therapies has been observed in hypermutated GBM caused specifically by *POLE* deficiencies (8, 9, 52, 53). An observational study showed that children with germline *POLD1* and *POLE* mutations exhibited durable objective responses in comparison with other mutational processes that also result in hypermutation (54). In addition, two pediatric patients with biallelic dMMR recurrent GBMs who exhibited exceptional and long-lasting responses to nivolumab harbored driver *POLE* mutations in their tumors (55). This is consistent with *POLE* mutations producing a distinct mutational signature based on the induction of specific patterns of nucleotide substitutions that generate neopeptides with increased hydrophobicity that are associated with response to ICB (50, 56). Notably, 35% of de novo hypermutated gliomas are associated with deficiencies in *POLE* (57), but this association is found in only 1% to 2% of all GBM tumors. For the majority of the 16.6% of GBMs that present as hypermutated at recurrence, this high-TMB phenotype is caused by the gaining of a dMMR phenotype that leads to tumor escape to the cytotoxicity of TMZ at more advanced stages (57, 58). Patients with TMZ-induced hypermutated GBMs do not experience a remarkable tumor T cell infiltration and did not show any improvement in survival after treatment with PD-1 blockade in a retrospective analysis (57).

TMB may lead to clonal (present throughout all tumor cells) or subclonal (present in a subset of tumor cells) neoantigens. In a series of 31 tumors from non–small cell lung cancer patients treated with pembrolizumab, only clonal and not subclonal neoantigens were recognized by T cells (59). Thus, clonal TMB is a critical driver of response to ICB as opposed to subclonal mutations (50, 59). In support of this, we reported that in the setting of immunotherapy, tumor clones with mutations that were predicted to lead to immunogenic neoantigens were present in pretreatment tumor samples, and were then specifically depleted in recurrent tumors of responder GBM patients treated with adjuvant PD-1 blockade (4). On the other hand, the determination of mutational signatures associated with response to ICB has the potential to provide a clinical benefit for a selected number of patients with GBM with high TMB. ICB response in GBM may also rely on other sources of antigens, such as transposable elements (60). Together, this evidence strongly indicates that the clonality and quality, but not quantity, of neoantigens are critical for immunotherapy efficacy (61), and suggests caution when considering use of only TMB as a biomarker for treatment of GBM with ICB.

### DNA replication stress and ICB response.

Beyond defects in dMMR, we recently identified that defects in DNA replication stress response may predict clinical outcomes to ICB in GBM and other nonhypermutated tumors (44). When DNA replication machinery encounters obstacles such as DNA lesions or atypical DNA structures that impede replication fork progression, cells activate the DNA replication stress response to stabilize the fork and ensure faithful genome duplication (62). However, this process is often dysregulated in cancer. We found that tumor cells with deficient DNA replication stress response have exhausted pools of replication protein A required to protect single-stranded DNA formed during replication stress, resulting in accumulation of immunostimulatory cytosolic DNA. Further, we found that a gene expression signature that can predict functional defects in the DNA replication stress response was associated with better outcomes in two studies evaluating PD-1 blockade in recurrent GBM from Zhao et al. (4) and Cloughesy et al. (5). This evidence suggests that PD-1 checkpoint inhibition might be beneficial for a particular subset of patients with GBM that could be identified by a unique molecular status involving DNA damage and replication stress, but validation in larger cohorts is needed for the implementation of this biomarker. These results also suggest that sensitivity to ICB may be induced by inhibitors of the key replication stress response kinases ATR and/or CHK1, which both have multiple compounds in varying stages of clinical trials.

### MAPK pathway activation and ICB response.

In an effort to distinguish those patients with GBM capable of exhibiting durable response to ICB as documented previously (1, 4, 63), we reported two clinical studies designed to identify molecular features associated with response to anti–PD-1 immunotherapy (4, 6). We and collaborators first reported that responder patients with recurrent GBM treated with adjuvant PD-1 blockade had enrichment of *BRAF* or *PTPN11* activating mutations in their tumors (odds ratio = 12.8) (4). However, the frequency of these mutations is very low among GBMs (2%–3%) (64), and these were found in only approximately 30% of responder patients. Thus, they are not a robust biomarker for identifying most patients who would experience benefit from PD-1 blockade.

Given that *BRAF*/*PTPN11* mutations induce signaling of the MAPK pathway, to develop a means of identifying responder patients who do not necessarily have MAPK activating mutations, through immunohistochemistry, we investigated the abundance of the phosphorylated/activated downstream effector ERK1/2 of the MAPK pathway (p-ERK) in recurrent GBM samples. p-ERK was present in tumors of patients with GBM exhibiting better clinical outcomes after adjuvant PD-1 blockade, but no difference was found in outcomes for patients who did not receive ICB (6). In other words, patients with high p-ERK levels in their tumors who underwent treatment with PD-1 exhibited longer overall survival than patients with low p-ERK levels, or than patients with elevated p-ERK staining who did not get immunotherapy. Yet, in patients not treated with immunotherapy, p-ERK was not associated with survival. All the responder patients had tumors with high p-ERK and included tumors that did not have *BRAF*/*PTPN11* mutations. Notably, most p-ERK staining derived from tumor cells. We observed a consistent association between p-ERK and survival in a second independent GBM cohort treated with adjuvant PD-1 blockade. Interestingly, elevated p-ERK was present in all responder patients, but not all patients with elevated p-ERK demonstrated prolonged survival (mean AUC of PD-1 blockade cohort: 0.78; mean AUC of no-immunotherapy cohort: 0.57). 

Interrogation of the immune microenvironment showed that patients with GBM showing better clinical outcomes had increased numbers of TMEM119^+^ microglial and Iba1^+^ myeloid cells in their tumors. By differential expression analysis using single-cell RNA-Seq, we showed that myeloid cells had enrichment of several inflammatory gene signatures, including “MHC class II protein complex binding” as the topmost expressed. Furthermore, MHC class II was abundantly expressed in myeloid cells from patients with GBM who had tumors with abundant p-ERK^+^ cells and longer survival after PD-1 blockade. In contrast, *PTEN*-mutated GBMs, which have poor responses to PD-1 blockade, had deficient MHC class II expression by CD68^+^ myeloid cells (4). In combination with previous reports suggesting immune evasion due to compromised MHC class II expression by glioma-associated microglia (65, 66), these results suggest that variations in MHC class II expression among GBM (19) may account for differences in clinical responses to immunotherapy (49). Furthermore, this shows the uniqueness of the tumor microenvironment in responder patients, consisting of an abundance of TMEM119^+^ microglial cells expressing MHC class II. Therefore, efficacy and biomarkers for response to immunotherapies for GBM point to a close relation with the antitumoral function of glioma-associated myeloid cells. In this regard, preclinical studies also highlight the important contribution of glioma-associated microglial cells to the efficacy of anti–PD-1 therapy (67).

Whereas p-ERK was found to be associated with survival and a different tumor immune microenvironment in patients with GBM treated with PD-1 blockade, additional steps are needed to implement this biomarker in the clinic. These include further validation in prospective studies, refinement of the cut point value for categorical decision making, and standardization of the technique to determine tissue sample quality, p-ERK epitope integrity, and consistency of assessment of p-ERK across different centers.

### Fc-γR polymorphisms and CTLA-4 ICB response.

Ongoing clinical trials are testing anti–CTLA-4 therapies in GBM, and therefore it is relevant to take into account the antitumoral mechanism of this immunotherapy. This form of ICB relies, in part, on T cell priming and depletion of intratumoral Tregs mediated by anti–CTLA-4 coengaging activating Fc-γ receptors (Fc-γRs), specifically CD16 (Fc-γRIIIA), expressed on antigen-presenting cells or natural killer cells, and CTLA-4 expressed on T cells (68). Interestingly, germline variants, i.e., single-nucleotide polymorphisms in *FCGR2A* (H131R) and *FCGR3A* (V158F), have been associated with improved outcomes owing to a higher binding affinity to IgG1 and IgG2, which increases antibody-dependent cellular cytotoxicity. These CD16 polymorphisms (V158F) have been shown to impact the response to ipilimumab, an IgG1 anti–CTLA-4 antibody, in patients with advanced melanoma (69). Among patients with high neoantigen burden, improved response rates were observed in patients who expressed the high-affinity polymorphic variant of CD16 as compared with those who only expressed the low-affinity variant (69). This is consistent with IgG1 anti–CTLA-4 antibodies demonstrating poor binding to the low-affinity CD16 receptor. These studies highlight that germline genetic variations in patients with cancer, such as Fc-γR polymorphisms, could contribute to effective immune activation, including efficient Treg depletion with anti–CTLA-4 therapies that would be relevant to assess in ongoing and future clinical trials testing this immunotherapy in patients with GBM.

**Figure 2:**
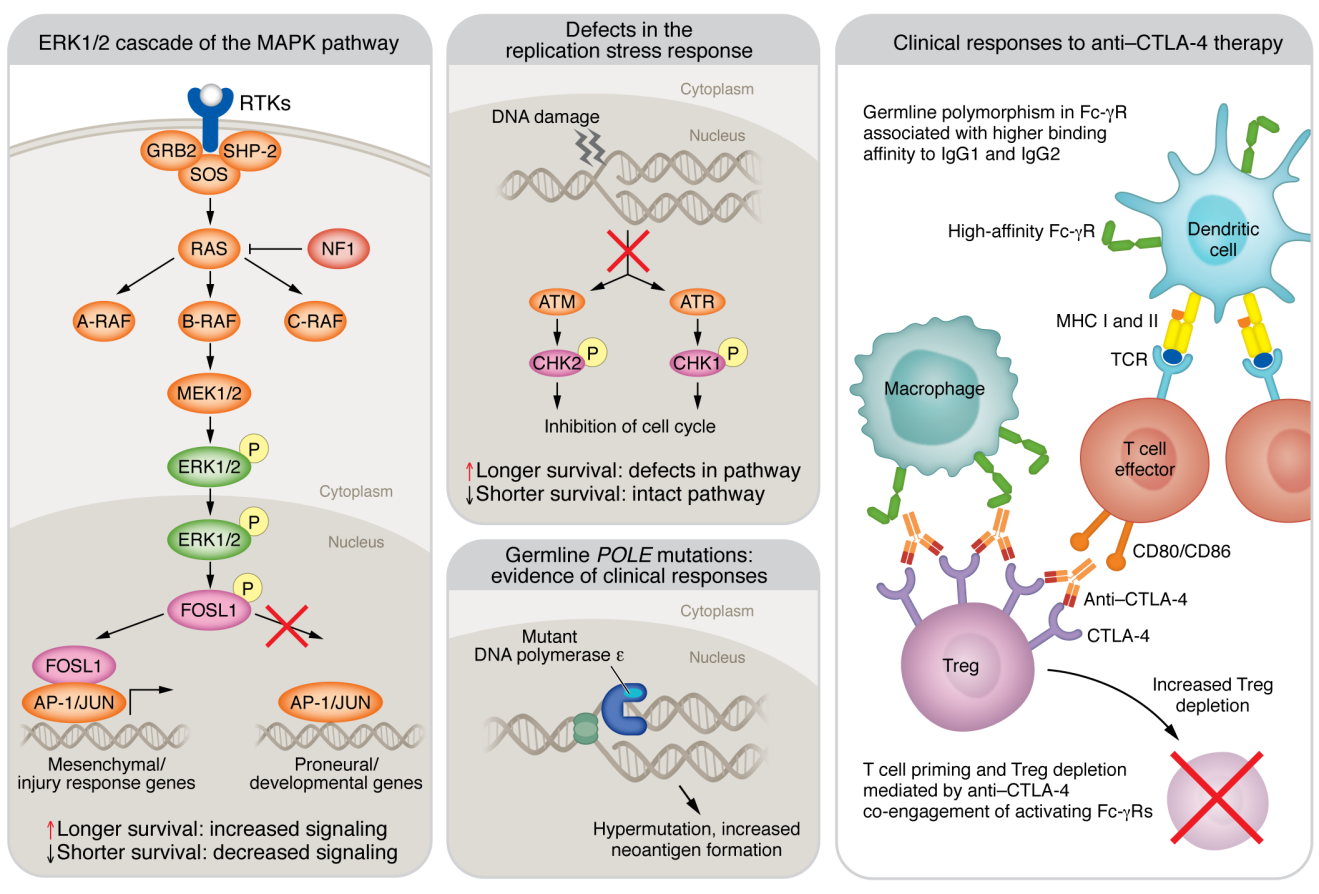
Summary of the potential mechanisms that might contribute to the response to ICB. Three potential mechanisms have been associated with better survival in patients with GBM treated with anti–PD-1 therapy: activation of the ERK1/2 cascade of the MAPK pathway; defects in the replication stress response in tumor cells; and germline *POLE* mutations. In addition, the mechanism underlying intratumoral Treg depletion is shown in the context of anti–CTLA-4 immunotherapy induced by antigen-dependent, cell-mediated cytotoxicity, which has been associated with germline polymorphisms of Fc-γR with high binding affinity to the therapeutic monoclonal antibodies. AP-1, activator protein 1; A-RAF, A-rapidly accelerated fibrosarcoma; ATM, ataxia telangiectasia mutated; ATR, ataxia telangiectasia and Rad3-related protein; CHK1, checkpoint kinase 1; FOSL1, Fos-related antigen 1; GRB2, growth factor receptor–bound protein 2; NF1, neurofibromin 1; SHP-2, Src homology region 2 domain–containing phosphatase-2; SOS, Son of Sevenless.

Assessing germline variants in GBM patients’ blood by sequencing and determining the transcript levels of Fc-γRIII in the tumor will be immediate steps necessary to investigate the relevance of Fc-γR polymorphisms with anti–CTLA-4 therapies in GBM. A summary of evidence highlighting the potential biomarkers for ICB in GBM is shown in Figure 2.

## The peripheral compartment in response to ICB

Cumulative clinical evidence accentuates the importance of peripheral immune cells as targets and drivers of response to ICB in cancer. Indeed, the peripheral immune compartment and secondary lymphoid organs are a central source of tumor-specific effector cells that expand and infiltrate tumor masses during treatment with PD-1 blockade (70, 71). Patients with brain metastasis exhibiting clinical responses after ICB illustrate the relevance of the generation of a peripheral immune response for intracranial tumors. Two phase II clinical trials evaluating ipilimumab and nivolumab in melanoma patients with untreated brain metastases and a trial of non–small cell lung cancer patients with brain metastases treated with pembrolizumab achieved complete and partial intracranial responses with this combinatorial strategy (72–74). Although these studies showed durable intracranial responses after treatment, there is no evidence linking clinical efficacy with a local generation of immune responses exclusively in the brain following immunotherapy. Instead, studies using preclinical models of brain metastases have shown that in the context of both anti–PD-1 and anti–CTLA-4 therapies, extracranial tumors and thus peripheral antigen stimulation are necessary to promote the expansion and trafficking of effector T cells into the brain as well as an increase in macrophages and microglia (75). These findings are also consistent with the great concordance between extracranial and intracranial responses in brain metastatic melanoma patients treated with ipilimumab and nivolumab. In addition, there was no evidence of intracranial beneficial responses with lack of extracranial progression (72, 73). In addition to a meaningful peripheral immune response during checkpoint blockade therapy, increased T cell numbers and cytolytic interactions with tumor cells might arbitrate successful intracranial responses following immunotherapy (43, 76). PD-1 expression was also found in myeloid-derived suppressor cells, CD11b^+^F4/80^+^ macrophages, and CD11c^+^MHCII^+^ dendritic cells in the tumor and the spleen of melanoma-bearing mice, albeit at different levels. Myeloid-specific PD-1 targeting induced antitumoral effects (77).

Further evidence implicating the peripheral compartment as a driver of responses to immune checkpoint inhibitors includes the ability of anti–PD-1 therapy to increase the proliferation of effector-like PD-1^+^ T cells in the peripheral blood (78). Contrary to conventional thinking that ICB therapy rescues exhausted T cells, it has been demonstrated that anti–PD-1 therapy induces the clonal replacement of tumor-specific T cells. By analysis of T cells by scRNA-Seq and T cell receptor (TCR) sequencing of pre- and post-immunotherapy tumor samples, rather than inducing the expansion and reactivation of preexisting exhausted tumor-infiltrating T cells toward an effector phenotype, PD-1 blockade induced the proliferation and tumor infiltration of peripheral tumor-specific T cell clones with a common phenotype that were not present before therapy in basal cell and squamous cell carcinomas (79). Furthermore, multiple studies have reported that a self-renewal PD-1^+^ T cell population with stem cell properties that expresses *TCF7* (coding for T cell factor 1) proliferates in the periphery and lymphoid organs and undergoes tumor antigen–driven expansion following anti–PD-1 therapy (79–83). Furthermore, exhausted TCF1 stem cell–like T cells have a better ability to control tumor growth compared with other T cell types, such as those that are terminally differentiated (81). The limited reinvigoration of tumor-infiltrating T cells and remodeling of the T cell landscape from the periphery after PD-1 blockade highlight the important function of peripheral reservoirs and potentially tertiary lymphoid structures that support the generation of antitumoral T cells. They also underscore the importance of other immunotherapies that promote T cell priming and CD8^+^ T cell memory formation, such as anti–CTLA-4, to further potentiate the generation of new tumor-reactive T cells (84). This needs to be considered within the context that tumor-intrinsic characteristics, in addition to iatrogenic chemotherapy and steroid utilization, modulate and limit the recruitment of T cells to the tumor microenvironment that suppresses the systemic immune reservoir.

In high-grade gliomas like GBM, patients can present with profound generalized lymphopenia (CD8^+^ and CD4^+^ T cells) that is further intensified by TMZ, radiotherapy, and steroids (85–87). In glioma patients, there are difficulties in the egress of T cells from the bone marrow to the peripheral blood, which is mediated by the loss of the sphingosine-1 type 1 receptor, critical for cell trafficking, on naive T cells (87). The T cells that are able to infiltrate gliomas have a hypofunctional phenotype demonstrated by low production of IFN-γ, IL-2, and TNF-α compared with matched and control PBMCs (88). The tumor-infiltrating lymphocyte (TIL) state of exhaustion is so profound in GBM that it cannot be sufficiently reversed (42). It is not expected that the therapeutic effects of immunotherapies would rely mainly on the reinvigoration of TILs, as ex vivo treatment with anti–PD-1 therapy shows limited reinvigoration of terminally differentiated CD8^+^ T cells isolated from human GBM (89). Considering the tumor-infiltrating T cell clonal replacement that occurs after anti–PD-1 therapy (79), treatment with ICB is expected to induce the expansion of specific T cell populations such as activated PD-1^+^ CD39^+^ T cells with potential tumor antigen specificity and increased TCR clonality found in the peripheral blood of glioma patients (90).

A recent clinical study of patients with GBM showed that neoadjuvant PD-1 blockade induced the expansion of *TCF7*^+^ progenitor exhausted T cells expressing markers of proliferation and cytolytic activity as well as *GZMK*^+^ T cells expressing *PDCD1* (PD-1), *HAVCR2* (TIM-3), and *IFNG* suggestive of activation and terminal differentiation with antigen specificity (12). Activated cytolytic T cells that expanded in the peripheral blood had TCR overlap with TILs in these patients with GBM. This was accompanied by increased intratumoral levels of T cells in patients with recurrent GBM treated with neoadjuvant PD-1 blockade compared with recurrent and newly diagnosed tumor patients who did not receive immunotherapy. As in other cancers, anti–PD-1 therapy induced the expansion of peripherally activated T cells that subsequently infiltrated the GBM microenvironment. Even though this evidence shows remodeling and increased levels of intratumoral T cells, it would be therapeutically relevant to determine whether supportive measures to replenish and revitalize the lymphoid compartment would improve clinical outcomes in lymphopenic GBM patients treated with neoadjuvant PD-1 blockade or other immunotherapies. The association of clinical responses with high number of baseline TILs seen in several cancers may reflect the ability of the peripheral immune system to resupply the tumor with new T cell clones during immune checkpoint inhibition in contrast to a reinvigoration of TILs (91).

As the peripheral compartment represents a critical component for therapeutic responses after ICB, variables assessing the immune fitness of the glioma patient’s immunity as well as a productive peripheral reaction during treatment could be clinically relevant as biomarkers of response to immunotherapies. For instance, baseline and on-treatment peripheral immune profiling and the assessment of adequate levels of immune cells and function of secondary lymphoid organs by surrogate markers might help identify and treat patients with GBM who would likely respond to immunotherapy. On the other hand, longer treatment with PD-1 inhibitors may support a delayed generation of key antitumoral T cell populations in GBM patients with lymphopenia. Evaluation of the innate and adaptive immune cell profile of T cells, B cells, natural killer cells, monocytes, dendritic cells, neutrophils, eosinophils, and basophils in both peripheral blood and tumor would provide insight into the phenotype and activation status of immune cells. This analysis has been used to assess baseline and therapy-mediated changes in local and peripheral cellular immunome in patients with pancreatic cancer, localized clear cell renal cell carcinoma, non–small cell lung cancer, and melanoma (92–96).

## Conclusions

We are at a critical juncture where the results of deep molecular and immune characterizations of gliomas need to be leveraged sufficiently to guide clinical management of patients with glioma tumors. Immunotherapies have the potential to change the clinical course of patients with GBM. While current benefit is limited to a small subset of patients, the use of biomarkers to guide immunotherapy would be a major step toward a personalized medicine approach, enable health care professionals to capitalize on the well-established reality of intertumoral heterogeneity in gliomas, and facilitate the development of the next generation of therapies and treatment paradigms. More complex biomarker tools have been developed in recent years with greater accuracy in predicting responses to therapies, in a multitude of cancers, including gliomas. We envision that a comprehensive panel of biomarkers will collectively improve our ability to identify patients most likely to benefit from immunotherapy and identify optimal treatment regimens that will improve outcomes for patients with GBM.

## Version 1 01/17/2023

Electronic publication
